# A telomere-to-telomere gapless genome assembly of the Tibetan wild ass (*Equus kiang*)

**DOI:** 10.1038/s41597-025-06494-4

**Published:** 2026-01-06

**Authors:** Bilige Wuyun, Jie Liu, Rihan Wu, Yunxia Li, Fangyuan Liu, Hengquan Zhao, Chunxia Hao, Gaoping Zhao, Wei Sun, Yongli Song, Wei Wang, Yu Wang, Cunxin Ma, Fengyi Xu, Jian He, Pengyue Wang, Xiangnan Bao, Guifang Cao, Yong Zhang, Ying Lu, Xihe Li

**Affiliations:** 1 State Key Laboratory of Reproductive Regulation & Breeding of Grassland Livestock, School of Life Sciences, Inner Mongolia University, Hohhot, 010020 China; 2 Research Center for Animal Genetic Resources of Mongolia Plateau, School of Life Sciences, Inner Mongolia University, Hohhot, 010020 China; 3 Inner Mongolia Saikexing, Institute of Breeding and Reproductive Biotechnology in Domestic Animal, Hohhot, 011517 China; 4 National Center of Technology Innovation for Dairy Industry, Hohhot, 010020 China; 5 Key Laboratory of Exploration and Utilization of Aquatic Genetic Resources, Ministry of Education, Shanghai Ocean University, Shanghai, 201306 China; 6 Key Laboratory of Animal Genetics, Breeding and Reproduction of Shanxi Province, College of Animal Science and Technology, Northwest A&F University, Yangling, Shanxi 712100 China; 7 Qilianshan National Park Qinghai Administration, Xining, 810000 China; 8 Key Laboratory of Animal Biotechnology of the Ministry of Agriculture, Northwest A&F University, Yangling, 712100 China

**Keywords:** Genome, Phylogenetics, Genome evolution

## Abstract

As a First-Class Protected species in China, the Tibetan wild ass (*Equus kiang*) is endemic to the Qinghai-Tibet Plateau and exhibits remarkable adaptation to extreme high-altitude environments characterized by low oxygen, cold temperatures, and high UV radiation. To explore its genetic basis and evolutionary adaptations, we generated the first telomere-to-telomere (T2T) gapless genome using an integrated sequencing strategy that combined PacBio HiFi, ultra-long Oxford Nanopore (ONT), Hi-C, and BGI short-read technologies. The resulting 2.49 Gb assembly achieved a contig N50 of 107.02 Mb, comprising all 54 telomeres and 27 centromeres across 25 pairs of autosomes and the X/Y chromosomes, and reached 99.94% BUSCO completeness. Genome annotation predicted 907 Mb of repetitive sequences (36.52% of the genome) and 24,005 protein-coding genes. Comparative and technical analyses confirmed high assembly continuity, completeness, and accuracy, with a consensus quality value (QV) of 64.73, providing a robust genomic reference for understanding the evolutionary mechanisms and adaptive traits of this iconic high-altitude ungulate.

## Background & Summary

The Tibetan wild ass (*Equus kiang*), a subspecies of the Asiatic wild ass (*E. hemionus*), inhabits the Qinghai–Tibetan Plateau, where it endures hypoxic, cold, and high-radiation conditions at elevations of 4,000–5,500 m^1^. Oxygen levels in these alpine regions are roughly 40% lower than at sea level^2^, while ultraviolet radiation is substantially higher^3^. Over time, this species has evolved physiological and genetic traits that support oxygen transport, cardiac performance, and skeletal development under chronic hypoxia^4^. *E. kiang* is endemic to the Qinghai–Tibetan Plateau, with most of its population residing in China, and is listed as a National First-Class Protected Species, with an estimated 60,000–70,000 individuals^1^.

Comparative genomics has deepened our understanding of *Equus* evolution. High-quality assemblies of the horse^5^, donkey^6^, Przewalski’s horse^7^, and plains zebra^8^ have revealed genetic variations linked to pigmentation, chromosomal stability, epigenetic regulation, and immune defense. Yet, how large-scale chromosomal organization contributes to species diversification within *Equus* remains unclear. To date, only the genomes of the domestic horse and donkey have achieved telomere-to-telomere (T2T) completeness, while a corresponding reference for the Tibetan wild ass has been lacking. The previously published Illumina-based draft genome was fragmented (2,960 scaffolds, scaffold N50 = 17.6 Mb) and lacked functional annotations (Genome Sequence Archive accession: CRA001222^4^), limiting its use for evolutionary and comparative analyses.

In this study, we present the first gapless, telomere-to-telomere genome assembly of the Tibetan wild ass, generated using PacBio HiFi, ultra-long Oxford Nanopore, BGI short-read, and RNA-seq data. This fully annotated reference provides the most complete genomic framework to date for this high-altitude equid. The availability of this T2T genome will enable chromosome-scale comparisons between *E. kiang* and its lowland relatives, advancing our understanding of high-altitude adaptation and informing future conservation efforts.

## Methods

### Ethical statement

This study was conducted under the approval of the Institutional Animal Care and Use Committee of Inner Mongolia University (Approval No. IMU-Horse-2024-150). All animal experiments followed both national and institutional regulations. Every effort was made to reduce animal discomfort and prevent unnecessary stress or injury.

### Sample collection

**Fig. 1:**
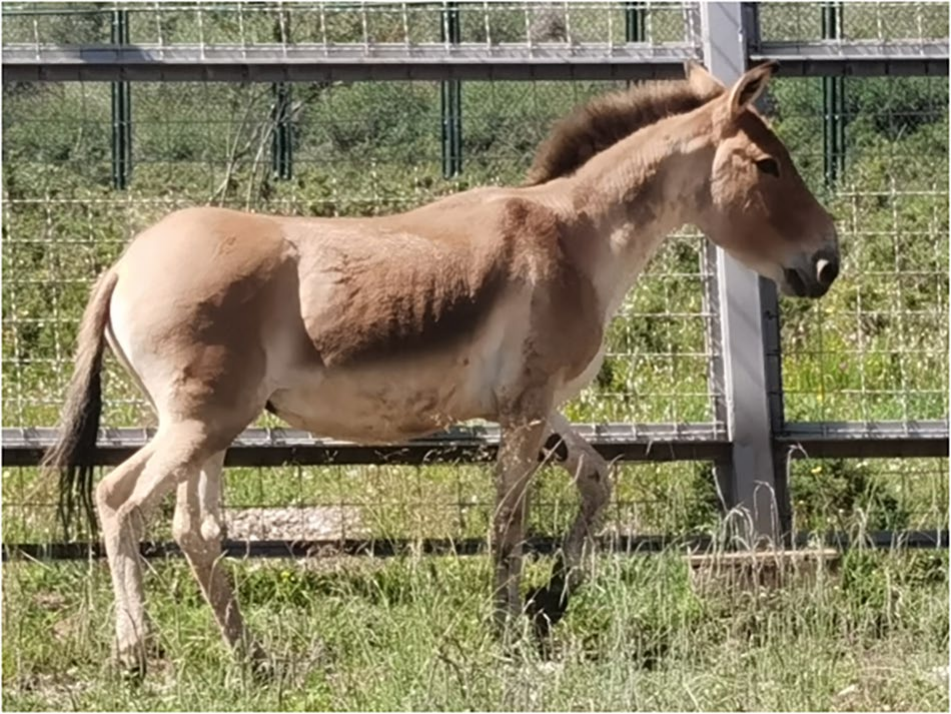
Image of the sequenced individual.

Fibroblast tissues were isolated from the ear of a 6-year-old male Tibetan wild ass (Fig. 1) in the Wildlife Rescue and Breeding Center of Qilian Mountain National Park, Qinghai, China (38°03′44″ N, 100°26′29″ E). The hair on the tissue surface was shaved before the sample collection and the area was disinfected with 75% ethanol (Cat. No. SJ2151, Lierkang). The tissues were then rinsed with 0.9% sodium chloride solution (Cat. No. 10139191323806, Kelun) to remove remaining blood and placed in Dulbecco’s phosphate-buffered saline (DPBS; Cat. No. C3590-0500, VivaCell) and transported to the laboratory at 4 °C, which were then used to establish the first fibroblast cell line of the Tibetan wild ass, which were verified by an immunofluorescence staining of Vimentin. The cells were cultured at 38.5 °C in a humidified incubator with 5% CO₂, using MEM-Alpha medium (Cat. No. 12561056, Gibco) supplemented with 10% fetal bovine serum (Cat. No. C04001-500, VivaCell) and penicillin-streptomycin (Cat. No. 15140-122, Gibco)^9^. When the cells exhibited stable growth and had been successfully passaged to the 4th-5th generation, sufficient samples were harvested for genome and transcriptome sequencing. We used the 4th-5th generations of the cells instead of primary cells because the primary cells are more prone to contamination and purity of the fibroblast cells is not high enough. Previous studies have reported that the fibroblast cells with 4-5 passages still maintain genomic stability and do not exhibit significant chromosomal or sequence-level variations, compared to the primary cells^10^.

### DNA extraction, library construction and sequencing

Genomic DNA was isolated from somatic cells using the sodium dodecyl sulfate-proteinase K (SDS-PK) method, which avoids toxic organic solvents such as phenol or chloroform and ensures to yield safety and high-quality DNA. High molecular weight genomic DNA (50–200 kb) for Oxford Nanopore Technologies (ONT) sequencing and genomic DNA (>30 kb) for PacBio circular consensus sequencing (CCS) were isolated using the Grandomics Genomic DNA Purification Kit (Cat No. XJZZ-HMW-001-003, GrandOmics, China) following the manufacturer’s standard protocol. DNA purity and quantity were determined with a NanoDrop™ One Microvolume UV-Vis Spectrophotometer (Thermo Fisher Scientific, USA) and a Qubit® 4.0 Fluorometer (Invitrogen, USA), respectively. Qubit measurements indicated concentrations of 330.4 ng/μL for Illumina short-read DNA libraries, 110.0 ng/μL for PacBio DNA libraries, and 158.0 ng/μL for ONT ultra-long DNA libraries. The OD260/280 ratios ranged from 1.8 to 2.0 and the OD260/230 ratios ranged from 2.0 to 2.2, indicating high-quality DNA suitable for long-read sequencing. DNA integrity was further verified by electrophoresis on a 1% agarose gel.

During the preparation of ONT sequencing library, large DNA fragments (>100 kb) were isolated and extracted using the Sage HLS system for high-molecular-weight DNA size selection (Sage Science, USA). DNA termini were subsequently repaired to convert heterogeneous blunt and sticky ends into a uniform state followed by dA-tailing, using the NEBNext FFPE DNA Repair Mix (Cat. No. M6630, New England Biolabs, USA) in combination with the NEBNext Ultra II End Repair/dA-Tailing Module (Cat. No. E7546, New England Biolabs, USA). Adapter ligation and purification were performed with the Ligation Sequencing Kit SQK-LSK114 (Oxford Nanopore Technologies, UK). The final sequencing library was quantified with a Qubit® 3.0 Fluorometer (Invitrogen, USA) and loaded onto the Oxford Nanopore PromethION platform (Oxford Nanopore Technologies, UK), which generated the raw sequencing data. When the reads were processed with Dorado in *sup* mode for quality control, those with a quality value (QV) < 7 were removed, producing 184.97 Gb of clean reads with an average length of 31.35 kb and an N50 length of 92.07 kb for downstream genome assembly (Table 1). In parallel, genomic DNA was sheared into 15–20 kb fragments using a Megaruptor 3 system (Diagenode) and purified for PacBio HiFi sequencing. Library construction was performed with the SMRTbell Prep Kit 3.0 (Pacific Biosciences, USA), followed by size selection and cleanup with the PippinHT system (Sage Science, USA). Sequencing on a PacBio Revio instrument (Pacific Biosciences, USA) operated by Grandomics (Wuhan, China) produced 145.03 Gb of data with a mean read length of 20.11 kb and an N50 of 21.36 kb. Additionally, a short-read library was prepared using the MGIEasy Universal DNA Library Prep Kit V1.0 (Cat. No. 1000005250, MGI) and sequenced on a DNBSEQ-T7RS platform (MGI, China), yielding 89.77 Gb of high-quality paired-end reads following quality control.

**Table 1:** Statistics of the sequencing data used in the genome assembly (len. for length).

Sequencing Platform	Total bases (bp)	Sequencing Depth (x)	Mean read len. (bp)	Maximum read len. (bp)	N50 read len. (bp)
ONT	184,970,760,484	74	31,345	1,161,957	92,072
PacBio HiFi	145,027,773,724	58	20,113	73,546	21,362
Hi-C	248,798,107,200	100	150	150	150
NGS data	89,767,184,400	36	150	150	150

The High-throughput Chromosome Conformation Capture (Hi-C) libraries were prepared from purified DNA digested with DpnII in 1 × NEB buffer. Blunt ends were filled in by adding 10 mM dTTP, 10 mM dCTP, 10 mM dGTP, 5 mM biotin-14-dATP, water, and 40 U of Klenow enzyme. Following ligation, crosslinks were reversed, and DNA was purified using the QIAamp DNA Mini Kit, then sheared to an average fragment size around 400 bp. Biotinylated junction fragments were captured with Dynabeads MyOne Streptavidin C1 beads, and libraries were constructed using the NEBNext Ultra II DNA Library Prep Kit. Sequencing was performed on an MGI-2000 platform, generating 248.80 Gb of clean reads after quality control (Table 1).

### RNA isolation and transcriptome-sequencing

Total RNA was extracted from the cell lines frozen in liquid nitrogen using TRIzol (Cat. No. DP424, TIANGEN) following the manufacturer’s protocol. Poly(A) RNA was enriched from total RNA using the Dynabeads mRNA Purification Kit (Cat. No. 61006, Invitrogen). Qubit analysis indicated RNA concentrations of 255.0 ng/μL for both short- and long-read RNA sequencing libraries. The long-read RNA sequencing library was prepared by first synthesizing full-length cDNA using an oligo(dT) primer and a template-switch oligonucleotide (TSO), followed by PCR amplification with barcoded primers to generate second-strand cDNA for Kinnex full-length RNA library construction. Sequencing of the long-read RNA library was performed on a PacBio Revio platform, yielding a total of 19.13 Gb of data, which enabled comprehensive characterization of full-length transcript structures. The short-read RNA sequencing library was constructed by purifying RNA with the Dynabeads mRNA Purification Kit, fragmenting it using the MGIEasy RNA Library Prep Kit V3.1 (Cat. No. 1000005276, MGI), and synthesizing first-strand cDNA with random primers and reverse transcriptase, followed by second-strand synthesis. The cDNA then underwent end repair, A-tailing, adapter ligation, and size selection using MGIEasy DNA Clean Beads (Cat. No. 1000005279, MGI), before PCR amplification to generate the transcriptome library. Sequencing on the MGI-T7 platform produced 7.6 Gb of paired-end reads, which were used for T2T genome annotation, allowing precise identification of exon boundaries and splice sites.

### Genome assembly

**Fig. 2:**
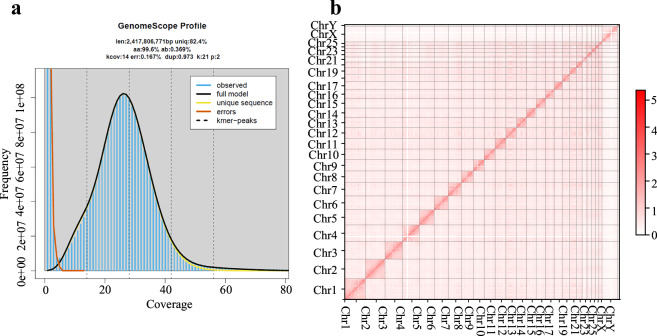
Genome features and Hi-C contact map. (**a**) GenomeScope plots of the 21-mer frequency. The curves in black indicate the distribution of the *k*-mer frequency. The red curves near the *y*-axis show the error of the *k*-mers, which normally occur at low coverage. The estimated genome sizes (Len) and heterozygosity rates (Het) are shown at the top of each plot, as is the percentage of uniquely mapped reads (Uniq), *k*-mer coverage (Kcov), PCR error rate (Err), percentage of PCR-derived duplicates (Dup), and *k*-mer length (K). (**b**) Hi-C contact map at 200 Kb resolution. Strength of intra- and inter-chromosomal Hi-C interaction frequencies is indicated by a color gradient scale displayed with strong interactions in red and weaker in lighter colors.

Prior to genome assembly, the depth and frequency distributions of 21-bp k-mers were analyzed with Jellyfish (v2.2.6)^11^ using 89.76 Gb of clean BGI reads (Table 1). GenomeScope2^12^ estimated the genome size at 2.42 Gb, with a low heterozygosity of 0.369% (Fig. 2a). Genome was *de novo* assembled with HiFiams (v0.19.5)^13^ using PacBio HiFi reads and ONT ultra long data (>50 kb) to generate a primary assembly, with a genome size of 2.56 Gb and a contig N50 value of 89.07 Mb.

The Hi-C reads were aligned to the assembled contigs using BWA (v0.7.17-r1198-dirty, mem -5SP)^14^, then filter the resulting BAM with filter_bam (v2.0.0, 1–nm 3)^15^, retaining only alignments with mapping quality ≥ 1 and edit distance < 3. Scaffold clustering was carried out successively by filtering, reassigning, ordering, and orienting with HapHiC (v1.0.5)^16^ according to the alignments. The clustered scaffolds were further manually curated based on Hi-C contact maps (Fig. 2b). The X and Y chromosomes were built from the phased assemblies by Hifiasm (v0.24.0)^17^ using Oxford Nanopore long reads in combination with Hi-C data (ONT and Hi-C). The sex chromosomes’ sequences were then incorporated into the Hi-C-based scaffolded genome, with the newly assembled Chr-X sequence replacing the original Chr-X in the scaffolds. The chromosome-scale genome is 2.50 Gb in size, with a scaffold N50 length of 107.20 Mb and 39 unclosed gaps.

The ONT ultra-long reads (>50 kb) were aligned to the genome using Minimap2 (v2.26, -ax map-ont)^18^. Reads that were either unmapped or exhibited poor alignment quality (sequence identity <80% and coverage <80%) and uniquely mapped to both flanking regions of a gap were extracted. These reads were subjected to local iterative assembly to generate sequences bridging the gap, which were then aligned back to the original genome and used to replace the corresponding gap regions. Similarly, PacBio HiFi reads were aligned to the genome using Minimap2 (v2.26, -ax map-hifi)^18^. Reads that were unmapped, contained telomeric sequences, or mapped to chromosome ends lacking assembled telomeres were extracted and subjected to local iterative assembly. The resulting sequences were aligned to the genome and used to replace the corresponding gap regions at chromosome termini. Thus, all the 39 gaps were successfully filled in this treatment.

**Fig. 3:**
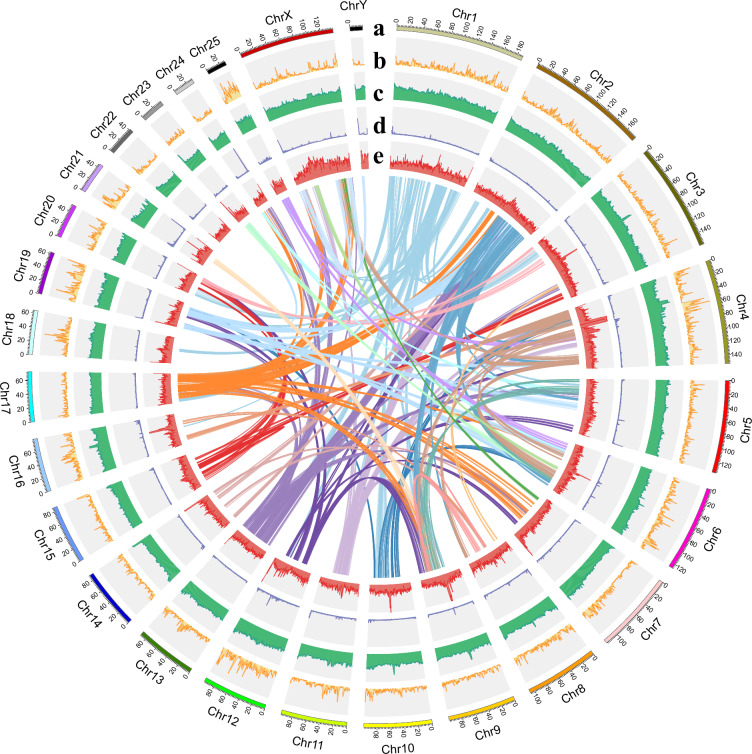
Genomic landscape calculated in 500-kb non-overlapping windows. The circles from outside to inside: (**a**) Length scale of each chromosome, (**b**) gene density, (**c**) GC content, (**d**) tandem repeats, (**e**) TE density. The lines in the central area of the circles link the syntenic chromosomes.

The gapless assemblies were polished using clean short reads to construct 21-kmer and 31-kmer databases with yak (v1.0; count -b37 -k21 and count -b37 -k31)^19^, followed by alignment of PacBio HiFi reads to the genome using minimap2 (v2.26,–secondary = no -ax map-hifi)^18^ and error correction with NextPolish2 (v0.2.0, default settings)^20^. Ultimately, a gapless T2T genome assembly was obtained, spanning 2.49 Gb with a contig N50 of 107.02 Mb, comprising 25 pairs of autosomes and the X/Y chromosomes (18.30–188.01 Mb in length), and exhibiting a GC content of 41.88% (Fig. 3 and Table 2).

**Table 2:** Overview of the assemblies.

Genome assembly	
Assembled genome size (bp)	2,486,009,346
Number of Chromosomes	52 (50 + XY)
Number of gap chromosomes	0
Number of gap centromeres	0
Length of anchored chromosomes (bp)	2,486,009,346
Length of non-anchored chromosomes (bp)	0
Contig N50 Length (bp)	107,027,462
Number of contigs	27
Number of telomeres	54
Number of centromeres	27
QV	64.73
Complete BUSCO (%)	99.94
**Gene prediction**	
Total length of annotated repetitive sequences (bp)	907,953,162
Percentage of repetitive sequences in the assemblies (%)	36.52
Quantity of annotated protein-coding genes	24,005
Average Gene Length (bp)	38,083
Average CDS length(bp)	1,531
Average Exons Number per Gene	8.74
Average Exon Length (bp)	175
Average intron length (bp)	4,724
BUSCO completeness (%)	94.66
**Quantity of predicted non-coding RNAs**	
rRNA	529
snRNA	490
miRNA	2,441
tRNA	2,103
Others	543

### Identification of telomeres and centromeres

**Fig. 4:**
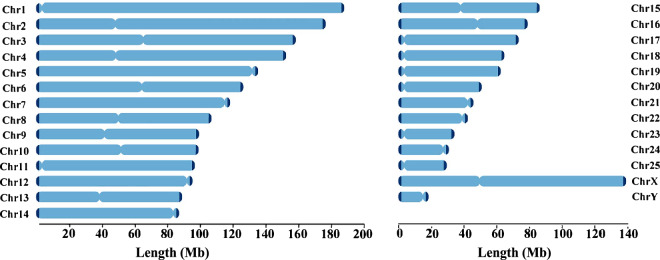
Location of centromeres and telomeres in the chromosomes. The filled blocks indicate the length of each chromosome, where deep blue and narrow areas exhibit the location of telomeres and centromeres, respectively.

Telomeres were annotated by scanning the terminal 100 kb of each chromosome in the T2T assembly with seqkit (v2.5.1, locate -i -p TelomereSeq)^21^, through which any region carrying ten consecutive AACCCT repeats was considered telomeric. Finally, telomeric sequences were identified at both ends of all the chromosomes (Fig. 3 and Table 3). Prediction of the centromere region involved the different tools. TRF (v4.09.1, parameters 2 7 7 80 10 50 500 -f -d -h -l 6)^22^ was employed to identify tandem repeats, after which RepeatMasker (v1.331, options nolow -no_is -gff -norna -engine abblast -lib lib)^23^ was used to annotate repetitive elements and extract regions of high repeat density. These high-density sequences were then used to build a k-mer database with KMC (v3.2.4; kmc followed by kmc_dump)^24^. SRF (-l 6)^25^ was then used to identify candidate centromere repeat units. Blastn (v2.9.0+)^26^ was applied to align candidate centromeric repeat units against the T2T genome and quarTeT (v1.1.6, CentroMiner -t 10 -i Genome–TE TE.gff)^27^ was used to predict centromere regions. In short, centromere regions were identified in all of the chromosomes in the T2T genome (Fig. 4 and Table 3).

**Table 3:** Location of telomeres and centromeres in the genome.

Chromosomes				Tolemeres		Centromeres	
Chr. ID	Len. (bp)	5p Start-End (bp)	5p Len. (bp)	3p Start-End (bp)	3p Len.(bp)	Start-End (bp)	Len. (bp)
Chr1	188,013,957	4-13131	13,128	188012009-188013951	1,943	64936-555010	490,074
Chr2	176,613,812	10-4457	4,448	176611231-176613761	2,531	47873361-48033270	159,909
Chr3	158,254,980	26-1884	1,859	158252879-158254889	2,011	63843373-66259309	2,415,936
Chr4	152,367,874	0-9650	9,651	152366799-152367873	1,075	44954817-51469249	6,514,432
Chr5	135,271,338	10-1726	1,717	135265349-135271336	5,988	135203297-135259476	56,179
Chr6	126,495,048	0-15354	15,355	126482218-126495047	12,830	63796011-64505598	709,587
Chr7	119,450,028	0-2407	2,408	119447896-119450027	2,132	117451604-118818646	1,367,042
Chr8	107,027,433	0-13516	13,517	107022900-107027432	4,504	49115124-50468229	1,353,105
Chr9	99,402,505	2-2170	2,169	99399142-99402504	3,363	39105552-43489663	4,384,111
Chr10	99,133,044	50-418	369	99131824-99133042	1,219	50627761-52196960	1,569,199
Chr11	96,649,823	30-5549	5,520	96632191-96649822	17,632	55506-270891	215,385
Chr12	95,826,724	50-9105	9,056	95822857-95826696	3,840	94480109-95770175	1,290,066
Chr13	89,048,944	50-4747	4,698	89046443-89048943	2,501	37700002-38849958	1,149,956
Chr14	88,180,332	3-1987	1,985	88176450-88180331	3,882	85469025-88176311	2,707,286
Chr15	86,144,096	1-1927	1,927	86138977-86144025	5,049	37210003-37949934	739,931
Chr16	78,732,551	0-1250	1,251	78722739-78732541	9,803	46237155-49517302	3,280,147
Chr17	73,072,830	0-2131	2,132	73054889-73072829	17,941	57714-95213	37,499
Chr18	64,458,441	26-8549	8,524	64454445-64458435	3,991	58488-378472	319,984
Chr19	62,705,790	95-4313	4,219	62699940-62705740	5,801	56030-1359745	1,303,715
Chr20	50,503,045	11-1426	1,416	50502646-50502847	202	53977-222700	168,723
Chr21	45,510,369	0-16393	16,394	45509512-45510368	857	45028293-45453002	424,709
Chr22	41,975,404	0-1383	1,384	41973956-41975403	1,448	41771427-41916672	145,245
Chr23	34,545,774	0-420	421	34543852-34545723	1,872	36016-1883012	1,846,996
Chr24	30,381,955	40-550	511	30377358-30381890	4,533	30163422-30325199	161,777
Chr25	29,224,820	0-10932	10,933	29221586-29224815	3,230	63158-525398	462,240
ChrX	138,721,515	0-19551	19,552	138706248-138721514	15,267	48970105-49470599	500,494
ChrY	18,296,835	0-14821	14,822	18281795-18296286	14,492	18113631-18139366	25,735

### Annotation of repetitive sequence

From the beginning, tandem repeats were predicted with GMATA (v2.2)^28^ and Tandem Repeats Finder (TRF, v4.07b)^22^. The LTR_retriever pipelines were then applied to build a curated LTR transposable-element library (TE-library), while RepeatModeler (open-1.0.11)^23^ generated a *de novo* repeat library (RepMod-library). The TE-library, the RepMod-library, and the Repbase database (v20181026) were merged into a comprehensive repeat library, which was subsequently used by RepeatMasker (Revision 1.331)^23^ to mask and annotate repetitive sequences across the entire genome. A total of 907 Mb (36.52% of the genome) was annotated as repetitive sequences, comprising LINEs (529.5 Mb), LTRs (182.8 Mb), SINEs (60.0 Mb), DNA transposons (96.2 Mb), tandem repeats (11.5 Mb), satellites (23.2 Mb), simple repeats (2.1 Mb), unknown elements (1.6 Mb), low-complexity sequences (0.06 Mb), and other minor categories (0.016 Mb) (Table 4).

**Table 4:** Statistics of repetitive sequence in the genome.

Type	Quantity	Length of sequence (bp)	Percentage of the genome (%)
Total TEs	2,771,155	869,496,718	34.98
LINE	1,169,958	529,459,787	21.30
LTR	584,979	182,827,255	7.35
SINE	391,126	60,026,886	2.41
DNA	613,536	96,235,675	3.87
RC	11,556	947,115	0.04
Tandem Repeats	326,057	11,474,999	0.46
Satellites	12,563	23,186,355	0.93
Simple repeats	11,795	2,076,892	0.08
Unknown	8,726	1,640,997	0.07
Low complexity	358	61,666	0
Others	175	15,535	0
**Sum**	**3,130,829**	**907,953,162**	**36.52**

### Prediction and annotation of the protein-coding genes

Gene prediction was performed using a combination of homology-based, transcriptome-based, and ab initio approaches. For homology-based annotation, GeMoMa (v1.6.1)^29^ was used to perform homology-based prediction by aligning against the protein sequences from horse (*E. caballus*, GCA_041296265.1), Przewalski’s horse (*E. przewalskii*, GCA_037783145.1), donkey (*E. asinus*, GCA_041296235.2), and plains zebra (*E. quagga*, GCA_021613505.1). For ab initio prediction, Augustus (v3.3.1)^30^ and GlimmerHMM (v3.0.4)^31^ were employed. Transcriptome-based annotation was performed by mapping RNA-seq clean reads to the T2T genome using STAR (v2.7.3a)^32^. Subsequently, transcript assemblies were integrated using PASA (v2.3.3)^29^, and gene loci were predicted based on transcriptome data with GeneMark-ST (v5.1, March 2014)^33^ and GMAP^34^. Furthermore, EvidenceModeler (EVM, v1.1.1)^35^ was employed to combine all evidence and generate a non-redundant consensus gene set. A total of 24,005 protein-coding genes were predicted in the T2T genome, with an average gene length of 38,082.92 bp and an average CDS length of 1,530.55 bp. On average, each gene contains 8.74 exons, with an average exon length of 175.16 bp and an average intron length of 4,723.89 bp. BUSCO analysis indicates a completeness of approximately 94.66% (Tables 2, 5).

**Table 5:** Prediction and functional annotation of the protein-coding genes in the genome.

Gene prediction							
Gene models		Quantity of genes	Average gene len. (bp)	Average CDS len. (bp)	Average exons number per gene	Average exon len. (bp)	Average intron len. (bp)
*De novo*	AUGUSTUS	27,830	36,994	1,639	8.44	194	4,754
	GLIMMER	184,371	12,278	660	4.66	141	3,172
*Homolog*	*E. asinus*	46,200	35,483	1,424	7.08	201	5,597
	*E. caballus*	47,666	35,881	1,449	7.29	199	5,477
	*E. przewalskii*	40,863	37,167	1,463	7.38	198	5,599
	*E. quagga*	40,499	37,354	1,464	7.41	197	5,595
RNAseq		115,648	29,249	3,513	6.11	575	5,035
Evidence Modeler		24,005	38,083	1,5315	8.74	175	4,724
**Functional annotation**							
**Database**		**Quantity of annotated genes**			**Percentage (%)**		
NR		21,480			89.48		
KEGG		15,375			64.05		
KOG		13,054			54.38		
GO		13,705			57.09		
Swissprot		19,633			81.79		
Annotated		21,678			90.31		

Functional annotation of the predicted genes was conducted by performing a Blastp (v2.7.1, with an *e*-value threshold of 1 × 10^−5^)^36^ search against several public databases, including the Non-Redundant Protein Database (NR), Kyoto Encyclopedia of Genes and Genomes (KEGG)^37^, Eukaryotic Orthologous Groups (KOG)^38^, Gene Ontology (GO)^39^, and SwissPro^40^. A total of 21,678 genes (90.31% of all predicted genes) were successfully annotated in at least one database. Specifically, 21,480 genes (89.48%) were annotated in NR, 15,375 (64.05%) in KEGG, 13,054 (54.38%) in KOG, 13,705 (57.09%) in GO, and 19,633 (81.79%) in SwissProt (Tables 2, 5).

Non-coding RNAs were annotated by aligning sequences to the Rfam (v14.0)^41^ database using RNAmmer and the cmscan program from the Infernal package^42^ to predict rRNA, snRNA, and miRNA genes. tRNA genes were identified using tRNAscan-SE^43^. A total of 529 rRNAs, 490 snRNAs, 2,441 miRNAs, and 2,103 tRNAs were predicted (Table 2).

## Data Records

All sequencing datasets generated in this study have been deposited in the National Center for Biotechnology Information (NCBI) under BioProject accession PRJNA1299250 (https://www.ncbi.nlm.nih.gov/bioproject/PRJNA1299250) and BioSample accession SAMN50296468 (https://www.ncbi.nlm.nih.gov/biosample/SAMN50296468). The sequencing data are publicly available in the SRA under the top-level accession SRP607874^44^, which includes the following read sets: SRR34937394, SRR34937391, SRR34937392, SRR34937393, SRR34937390, and SRR34997386. These datasets comprise PacBio HiFi sequencing, ONT ultra-long reads, short-read Hi-C data, next-generation sequencing data, and RNA-Seq datasets. The T2T genome assembly has been deposited in the NCBI Genome database under accession GCA_052150635.1^45^. The gene structure annotation file^46^, coding sequences^47^, and protein sequences^48^ have been deposited in Figshare under the following accession numbers: 10.6084/m9.figshare.299415144, 10.6084/m9.figshare.29941520, and 10.6084/m9.figshare.29941517, respectively. All data are publicly available.

## Technical Validation

**Fig. 5:**
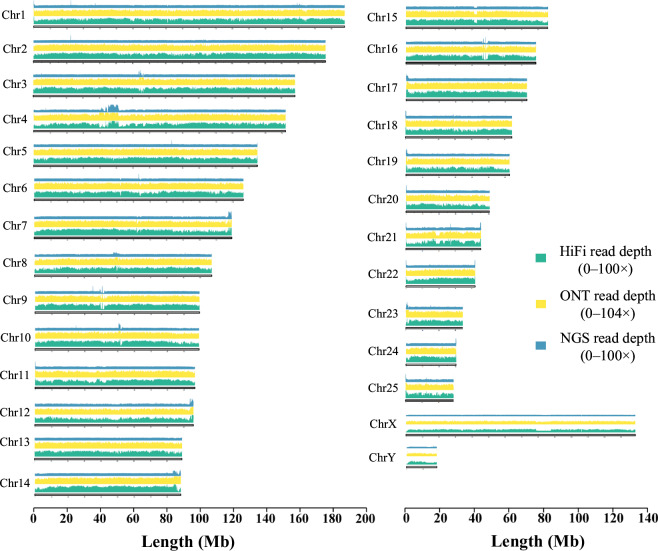
Sequencing coverage of Hi-C, ONT, and NGS reads on each chromosome. Coverage depth is plotted using 50-kb bins.

Multiple complementary strategies were applied to assess the completeness and accuracy of the genome assemblies. Hi-C contact maps displayed strong intra-chromosomal interactions across all linkage groups, confirming the correct ordering and orientation of contigs as well as the overall chromosomal architecture (Fig. 2b). Annotation of centromeric regions identified 27 centromeres on 25 autosomes and 2 sex chromosomes, each corresponding to an individual chromosome, while telomeric sequences were detected at both ends of all chromosomes, further supporting assembly integrity (Fig. 4). Short paired-end reads were aligned using BWA (v0.7.17)^14^, resulting in a mapping rate of 99.38% for BGI reads and 96.58% for RNA-Seq reads. Long-read alignments performed with minimap2^49^ also demonstrated high mapping efficiencies, with 95.76% of ONT reads and 99.99% of PacBio HiFi reads successfully mapping to the T2T genome (Fig. 5).

**Fig. 6:**
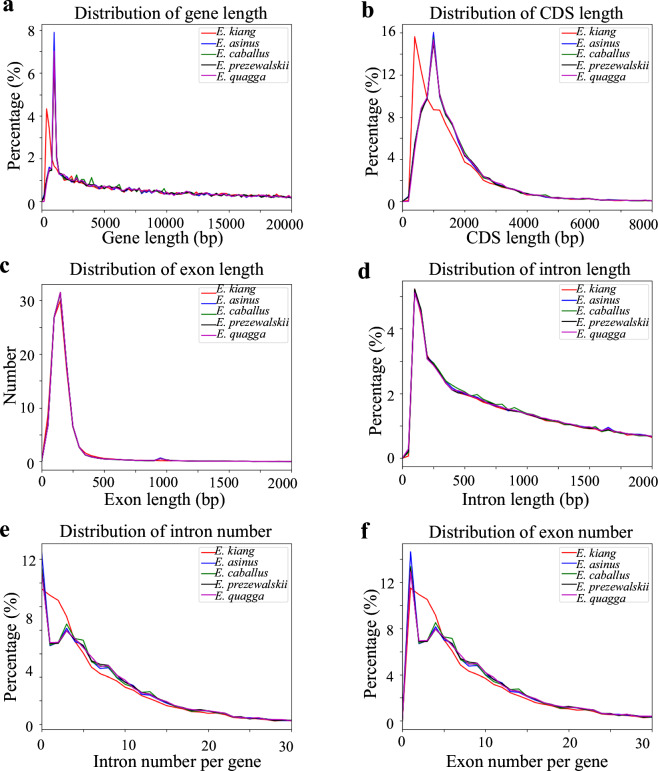
Distribution of the genome features among different species. (**a**) Distribution of gene length. (**b**) Distribution of CDS length. (**c**) Distribution of exon length. (**d**) Distribution of intron length. (**e**) Distribution of intron numbers per gene. (**f**) Distribution of exon numbers per gene.

Comparative analyses of gene feature distributions (such as gene counts, CDS regions, exons, and introns) between the Tibetan wild ass and closely-related species revealed consistent patterns, supporting the accuracy of gene annotation (Fig. 6). CRAQ^50^ evaluation of the assembly indicated a high coverage rate of 99.29%, with only 0.032% of regions classified as low-confidence, and near-perfect assembly quality indices for reads (R-AQI: 99.17) and scaffolds (S-AQI: 98.69), reflecting excellent continuity and base-level precision. Merqury (v1.3)^51^, based on a 21-mer database constructed with Merfin, estimated the consensus quality value (QV) at 53.74 for BGI short reads (overall error rate: 4.23 × 10^−6^) and 64.73 for PacBio HiFi reads (overall error rate: 3.37 × 10^−7^), confirming exceptional base-level accuracy. Additionally, BUSCO test^52^ using compleasm (v0.2.5)^53^ with the mammalia_odb10 dataset^54^ of 9,226 single-copy orthologs confirmed a genome completeness of 99.94%.

Overall, the validation of the genome data set demonstrated that the current T2T genome of the Tibetan wild ass was highly completed and structurally reliable, which established a robust reference for investigating the evolutionary and adaptive mechanisms of hoofed mammal in high-altitude environments.

## Data Availability

All sequencing data generated in this study have been deposited in the National Center for Biotechnology Information (NCBI) under the BioProject accession PRJNA1299250 and BioSample accession SAMN50296468. The sequencing datasets are also available in the Sequence Read Archive (SRA) under the top-level accession SRP60787446. These include PacBio HiFi and ONT ultra-long read data (BAM format; SRA accessions SRR34937394, SRR34937393, short-read Hi-C and next-generation sequencing data (FASTQ format; SRA accessions SRR34937392, SRR34937391, as well as RNA-Seq datasets obtained from both PacBio HiFi and short-read sequencing (FASTQ format; SRA accessions SRR34997386, SRR34937390. The T2T genome assembly has been deposited in GenBank under accession GCA_052150635.1 (FASTA format; https://ftp.ncbi.nlm.nih.gov/genomes/all/GCA/052/150/635/GCA_052150635.1_ASM5215063v1/GCA_052150635.1_ASM5215063v1_genomic.fna.gz). Gene structure annotation (GFF format), coding sequences (FASTA format), and protein sequences (FASTA format) are available from the Figshare database under the following DOIs: 10.6084/m9.figshare.29941514.v1, 10.6084/m9.figshare.29941520.v1, and 10.6084/m9.figshare.29941517.v1. All data are publicly accessible.

## Code availability

All data processing was conducted according to the standard protocols and manuals of the respective bioinformatics tools, with no custom scripts developed for this study.
